# Ibuprofen During the COVID-19 Pandemic: Social Media Precautions and Implications

**DOI:** 10.5811/westjem.2020.4.47686

**Published:** 2020-04-24

**Authors:** Brandon M. Carius, Steven G. Schauer

**Affiliations:** * Brooke Army Medical Center, JBSA Fort Sam Houston, Texas; † US Army Institute of Surgical Research, JBSA Fort Sam Houston, Texas; ‡ Uniformed Services University of the Health Sciences, Bethesda, Maryland

The ongoing spread of COVID-19, also known as the novel coronavirus, has created significant concerns often leading to panic throughout the world as to its virulence and lethality. Regularly published media track newly infected patient rates and deaths further driving public panic, which invariably leads to people seeking information. As the use of social media continues to complement and augment the drive for free, open-access medical education, some have previously highlighted limitations posed by such a largely unregulated online venue.^1^ Previously, these opinions targeted much smaller populations, such as the push against electronic cigarettes after vaping-associated pulmonary injury was identified in 2019. With the increasing reach of COVID-19, however, concerns are not isolated to one section of society, but have instead permeated widely.

The potential for danger and patient harm is already evident, even as the United States and the world begin to grapple with large-scale lockdowns and nationwide efforts. The recent cascade of events surrounding the use of ibuprofen to treat fever in COVID-19 patients illustrates the power and deception of social media. On March 14, French Health Minister and physician Olivier Verán tweeted that “taking anti-inflammatory drugs (ibuprofen, cortisone …) could be an aggravating factor of the infection.” Within 24 hours, over 43,000 individuals retweeted this advice despite little evidence to support this claim. In this short time, the World Health Organization’s (WHO) Christian Lindmeier, when asked about the tweet, quickly agreed that “we recommend using paracetamol, and do not use ibuprofen as a self-medication.”^2^ Major international news organizations immediately were abuzz with stories regarding the dangers of ibuprofen in COVID-19, “#ibuprofen” trended on social media, with many declaring it should not be used to treat fever at all. Twenty-four hours later, the official push against ibuprofen seemed to suddenly halt, and the WHO declared that it would not recommend against the use of ibuprofen to treat medical conditions, including COVID-19 patients.^3^

All of this concern was driven by a single letter published online in the *Lancet* on March 11 stating that the use of ibuprofen could theoretically increase the expression of angiotension-converting enzyme 2 (ACE2) and facilitate COVID-19 infection.^4^ It is important to note that this letter cited no studies to directly link ibuprofen use to ACE2 expression. Yet with a single tweet, we witnessed a major ripple effect against the use of a foundational antipyretic, and within 24 hours a whiplash back. But, there is no way to discern just how far the original statements reached, or whether the subsequent recalls undid the original misinformation entirely. While Dr. Verán’s statement was retweeted thousands of times, it remains unclear how far this information penetrated given that recalls rarely carry the impact that original statements do.

Similar social media turbulence has occurred with hydroxychloroquine and chloroquine, treatments for malaria and rheumatological diseases that have shown initial promise in small trials. This led to scarcity concerns that groups would try to purchase large quantities preemptively in case of approval by the US Food and Drug Administration (FDA).^5^ Some states then went as far as to restrict prescription practices specifically out of concern for stockpiling these medications.^6^ This further demonstrates the effect that social media have on the practice of medicine even in the absence of science supporting these practices. Never have we witnessed such a large-scale concern over a generic medication in the setting of a health crisis.

While news organizations continue to search for up-to-the-minute stories, and seek the most page views with sensational headlines, medical professionals must recognize our role in providing unsensational care. Efforts should be made to funnel questions to official organizations, such as the Centers for Disease Control and Prevention, the FDA, and local healthcare networks. Patients with concerns should seek evaluation and care in the clinical setting, not through social media. The turbulence of declarations and retractions makes the unease of this time all the worse. We must help centralize information by refraining from declarative diatribes and pushing unproven correlations.
